# Dermoscopy of Aplasia Cutis Congenita: A Case Report and Review of the Literature

**DOI:** 10.5826/dpc.1101a154

**Published:** 2021-01-29

**Authors:** Rasna Neelam, Mio Nakamura, Trilokraj Tejasvi

**Affiliations:** 1 University of Michigan Medical School, Ann Arbor, MI, USA; 2 Department of Dermatology, University of Michigan, Ann Arbor, MI, USA

**Keywords:** aplasia cutis congenita, alopecia, dermoscopy, trichoscopy

## Introduction

Aplasia cutis congenita (ACC) is a rare heterogeneous congenital disorder characterized by focal or widespread absence of the skin.

Its estimated incidence is 1–3 in 10,000 live births and about 70% to 90% of cases present as isolated scalp lesions [1]. The condition can present variably depending on the tissue layers involved and the extent of healing in utero. Commonly, ACC presents as a small, solitary hairless skin defect covered with atrophic tissue, eschar, granulation tissue, or ulcerations. It can be classified into membranous and non-membranous clinical subtypes and has also been categorized into 9 groups based on location and associated congenital anomalies [1]. Herein, we describe a case of ACC diagnosed by using dermoscopy as a helpful diagnostic tool.

## Case Presentation

**Figure 1:**
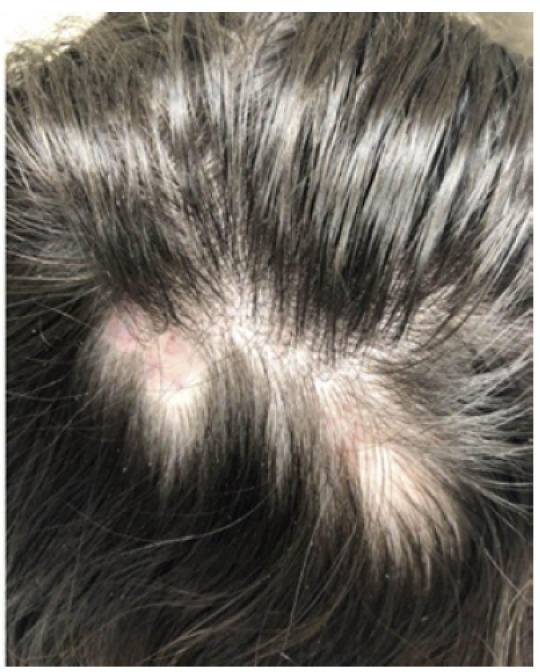
An atrophic white patch of alopecia on the scalp

A woman in her early twenties with a history of alopecia since birth presented with 2–3 months of waxing and waning, dull, throbbing, and point tenderness in one of the patches of alopecia. The alopecic patch had been present on the right parietal-occipital scalp since birth and has been stable in size and appearance for years. Three other round patches of alopecia had also been present since birth and remained asymptomatic. On clinical examination of the scalp, an atrophic white patch of alopecia was noted (Figure 1). Ill-defined bogginess was noted surrounding the lesion, extending to the central and posterior occipital scalp. The differential diagnosis included ACC, alopecia areata, tinea capitis, and trichotillomania.

**Figure 2:**
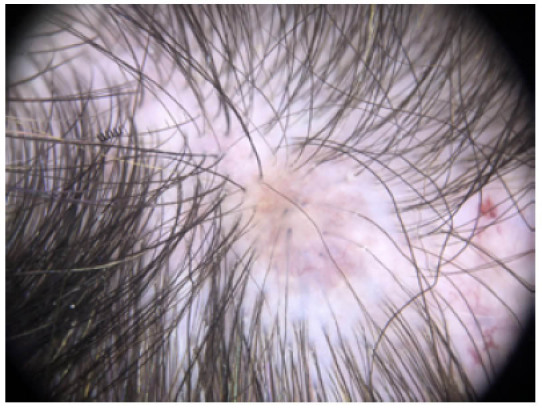
Dermoscopy of the alopecic patch shows a translucent epidermis with multiple branched blood vessels and hair bulbs at the edge of the patch.

On dermoscopic examination, the alopecic patch showed a translucent epidermis with multiple branched blood vessels and hair bulbs at the edge of the patch (Figure 2).

Based on the clinical and dermoscopic findings, the diagnosis of ACC was favored. A non-contrast head CT showed no evidence of osseous, vascular, neural, or soft tissue defects. The patient was counseled to avoid trauma to the lesion and was instructed to follow up with her primary care physician for management of scalp tenderness and pain, likely representing headache or migraine, rather than a symptom of the lesion.

## Conclusions

Recent published reports demonstrate the emerging use of dermoscopy and trichoscopy as a valuable tool for diagnosing ACC [2–5]. Dermoscopic images at hair-bearing margins reveal a translucent epidermis, a visible vascular network, and hair bulbs arranged radially along the margins of alopecia [2,3]. Additional dermoscopic findings have included absent follicular openings, thicker vessels, and distinct collar hypertrichosis [2]. Hair bulbs have been shown on trichoscopy, visible through a semi-translucent epidermis [3]. Radially oriented, horizontally extending hair follicles have been described as starburst-like [4] or “golf club set-like” [5] and are clearly visible from the bulbs to the follicular ostia under a thin epidermis. As noted above, on our dermoscopic evaluation, we noted a translucent epidermis, visible vascular network, and radially arranged hair bulbs. These specific findings are consistent with ACC.

Dermoscopy is useful in distinguishing ACC from other similar appearing disorders in its differential such as alopecia, tinea capitis, and trichotillomania. ACC can be differentiated from other types of congenital or acquired alopecia, such as triangular temporal alopecia, which shows presence of vellus hairs only in affected sites; alopecia areata, which shows black dots, yellow dots, and exclamation hairs; tinea capitis, which shows coiled or comma shaped hairs; and trichotillomania, which shows broken hairs and flame hairs [3]. Membranous ACC can also be distinguished from nevus sebaceous by visualizing a lack of skin appendages and a translucent appearance [2]. While biopsy for histopathologic evaluation can be performed to diagnose ACC, showing a thin layer of dermal collagen without epidermis or adnexal structures, biopsies can be avoided if the diagnosis can be made clinically.

Currently, there is no consensus on the management of ACC. Conservative therapy is generally suggested for small lesions, whereas the risks and benefits of surgical intervention are often weighed for treatment of larger lesions in consultation with pediatric plastic surgeons.

In conclusion, dermoscopy/trichoscopy is a valuable diagnostic tool for ACC and can help distinguish ACC from other types of congenital or acquired alopecias. Dermatologists should familiarize themselves with the dermoscopic findings of ACC—a translucent epidermis, a visible vascular network, lack of appendages, and hair bulbs arranged radially along or sometimes within the margins of alopecia.
